# Accelerated Optimized Protocol of Intermittent Theta-Burst Stimulation for Negative Symptoms in Schizophrenia (ACTh-NS): A Randomized, Double-Blind, Sham-Controlled Study Design

**DOI:** 10.3390/brainsci15091021

**Published:** 2025-09-22

**Authors:** Ivan Taiar, July Silveira Gomes, Lucas Jorge, Carolina Ziebold, André Fernandes, Renan Biokino, Pedro Lorencetti, André Brunoni, Ary Gadelha

**Affiliations:** 1 Interdisciplinary Laboratory of Clinical Neurosciences (LINC), Department of Psychiatry, Paulista School of Medicine, Federal University of São Paulo (EPM/UNIFESP), São Paulo 04017-030, Brazil; ivan.taiar@unifesp.br (I.T.); giorgini.lucas@outlook.com (L.J.); carolaziebold@gmail.com (C.Z.); afsilva12@unifesp.br (A.F.); renanbiokino@gmail.com (R.B.); plorencetti@gmail.com (P.L.); aryararipe@gmail.com (A.G.); 2 Department and Institute of Psychiatry, Medical School from São Paulo University, Sao Paulo 01246-903, Brazil; brunoni@usp.br

**Keywords:** schizophrenia, negative symptoms, transcranial magnetic stimulation

## Abstract

Introduction: Intermittent theta burst stimulation (iTBS) has been associated with improvements in the negative symptoms (NSs) of schizophrenia. However, optimizing by shorter protocols remains necessary. Furthermore, understanding their impact on other clinical symptoms, sleep, and autonomic regulation is important to underlying therapeutic effects. Objectives: Evaluate the efficacy of an accelerated iTBS protocol on reducing NSs in patients with schizophrenia. We hypothesize a 20% reduction in BNSS scores in the active group, as well as improvements in disorder-related aspects, including sleep patterns, symptoms severity, and cognition. Methods: A double-blind, randomized, sham-controlled clinical trial design will be conducted to test the effects of the accelerated iTBS protocol in 60 participants with schizophrenia (30 active and 30 sham) with moderate NSs. iTBS protocol will consist of four daily sessions, with 600 pulses per session for five consecutive days. Patients will be assessed at three time points (baseline, after intervention and 30 days follow up) for clinical symptoms, cognition and heart rate variability. The primary outcome will be negative symptoms using the Brief Negative Symptom Scale (BNSS). Study register: Brazilian Registry of Clinical Trials (CAEE: 71102823.4.0000.5505). Conclusions: The accelerated iTBS protocol has demonstrated promising effects on NSs. However, it is still necessary to establish an effective and feasible high-dosage protocol. This study will contribute to optimizing therapeutic protocols for schizophrenia, with a particular focus on clinical applicability. Additionally, it will provide an opportunity to deepen the understanding of the physiological effects of neuromodulation, contributing to the understanding of its underlying mechanisms.

## 1. Introduction

Schizophrenia is a mental disorder that affects approximately 0.4% of the population and is characterized by a complex and heterogeneous behavioral and cognitive syndrome, resulting from brain developmental abnormalities caused by the interaction of genetic risk factors and environmental exposures [1]. Despite its low prevalence, the disorder typically leads to substantial impairments related to quality of life, patient functionality, and prognosis, even with the best available treatments [2]. Although negative symptoms (NSs) are among the hallmark features of schizophrenia with significant clinical relevance, these symptoms present challenging for treatment and lack targeted therapeutic options. NSs are the primary predictor of low functionality and may be associated with impaired cognitive performance in these patients [3].

Neuromodulation techniques have emerged as promising alternatives for the treatment of negative symptoms. Non-invasive brain stimulation (NIBS) encompasses non-pharmacological techniques, either electrically or magnetically induced, that are frequently used in psychiatric disorders [4,5]. In this context, transcranial magnetic stimulation (TMS) has been distinguished as a technique that is capable of stimulating the cerebral cortex through a focal magnetic field, which induces an electrical current in the underlying brain tissue, promoting changes in cortical excitability and neuronal function [6,7]. In 2018, the FDA approved a new TMS modality, theta-burst stimulation (TBS), which delivers magnetic pulses in a specific “burst” pattern that can mimic endogenous theta rhythms, allowing treatments to be administered within a few minutes [8,9,10]. TBS protocols present significant advantages compared to traditional rTMS due to their shorter duration, enabling accelerated protocols in which multiple TBS sessions can be administered per day, delivering higher pulse doses within shorter periods with clinical safety and efficacy [4].

A recent meta-analysis that included 12 intervention studies using intermittent theta burst stimulation (iTBS) for negative symptoms showed that a higher total number of pulses, compared to relatively lower numbers of pulses, presents a greater effect size, suggesting that the efficacy of iTBS treatment is dose-dependent [11]. Definitions of parameters such as intensity, frequency, duration, waveform, and stimulation site need to be better defined. In this regard, accelerated TMS (aTMS) protocols, characterized by two or more sessions of TMS in a single day, have emerged as an effective alternative and appear to be a viable strategy for reducing total treatment time; it enables a greater number of sessions per day and delivers the same number of pulses and sessions of a conventional 6-week treatment within a single week, sometimes with efficacy surpassing that of daily session doses [12,13,14]. Despite its potential, the literature about the use of intermittent theta burst stimulation (iTBS) for treating negative symptoms of schizophrenia is still limited, especially regarding the type of protocol and the brain area to be stimulated, although the left dorsolateral prefrontal cortex (DLPFC) has shown promising results [15,16,17,18,19].

The studies conducted to date have limitations that hinder a better understanding of the true potential of intermittent theta burst stimulation (iTBS) for negative symptoms in schizophrenia, including the following: 1—few double-blind, randomized clinical trials; 2—small sample sizes; 3—the use of instruments that do not allow for the assessment of sub-domains of negative symptoms; 4—a lack of control for potential confounders in the evaluation of negative symptoms, such as depressive and extrapyramidal symptoms [15,16,17,20,21].

In addition to clinical improvement, the use of TMS has investigated the impact on physiological variables, among which is heart rate variability (HRV), a biomarker of autonomic nervous system activity and the brain–heart interaction through vagus nerve activity [22]. Physiological self-regulation is an adaptive process that is directly connected to volition and cognitive functioning in the general population [23], and it is shown to be reduced in various disorders [24], including schizophrenia [25], where dysregulation is observed regardless of antipsychotic use [26,27,28,29,30]. Research conducted with this population has not shown differences in blood pressure modulation and heart rate following rTMS; however, variables related to HRV associated with the fronto-vagal network were not analyzed [31].

Sleep disturbances are frequent in schizophrenia and go beyond pharmacological or psychotic-state effects. In patients with negative symptoms, they have been closely linked to clinical features of the disorder, including an association between an altered sleep architecture with prefrontal dysfunction, aggravating motivational deficits, social withdrawal, and cognitive impairments [32]. Autonomic nervous system dysregulation is reported in association with sleep disturbances in several pathologies [33,34], with hyperarousal states during sleep being associated with predominant sympathetic activity [33] and parasympathetic predominance being reported in association with some hypersomnia disorders [34].

In this context, a clinical trial using an accelerated iTBS protocol in the left dorsolateral prefrontal cortex (DLPFC) is necessary, considering the positive results from studies conducted with conventional high-frequency rTMS protocols and the fact that accelerated protocols using iTBS have been demonstrated to be more effective [35]. The primary objective of the study will be to evaluate the efficacy of an accelerated transcranial magnetic stimulation (theta-burst—iTBS) protocol on reducing negative symptoms in patients with schizophrenia through a double-blind, randomized, sham-controlled clinical trial. The protocol aims to provide a greater number of sessions in a short period of time while maintaining the feasibility of application. The study will include multimodal assessments, including clinical, neuropsychological, sleep, and HRV evaluations, in order to establish the mechanisms of stabilization of these symptoms. We hypothesize a 20% reduction in BNSS scores in the active group, as well as improvements in disorder-related aspects, including sleep patterns, symptom severity, and cognition.

## 2. Materials and Methods

**Figure 1:**
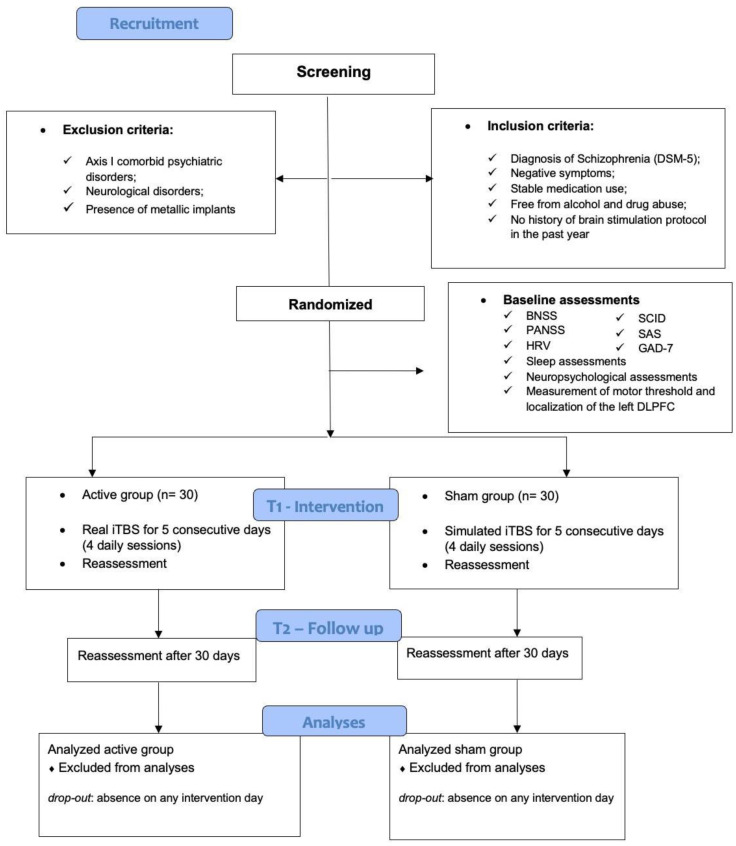
CONSORT flowchart of the recruitment process. DSM-5 (Diagnostic and Statistical Manual of Mental Disorders, Fifth Edition), BNSS (Brief Negative Symptom Scale), PANSS (Positive and Negative Syndrome Scale), HRV (hear rate variability), SCID (Structured Clinical Interview for DSM Disorders), SAS (Simpson-Angus Scale), GAD-7 (Generalized Anxiety Disorder), DLPFC (Dorsolateral Prefrontal Cortex), iTBS (Intermittent Theta-Burst Stimulation), T1 (timepoint 1), T2 (timepoint 2).

Design: We proposed a randomized, double-blind, sham-controlled clinical trial to test the effects of the accelerated iTBS protocol in participants with schizophrenia with negative symptoms (CONSORT—Figure 1). The study is conducted in accordance with the Declaration of Helsinki and has been approved by the Research Ethics Committee of the Federal University of São Paulo (UNIFESP) as of 14 September 2023 and registered in The Brazilian Registry of Clinical Trials (ReBEC) (CAEE: 71102823.4.0000.5505).

*Participants*:

We will recruit 60 volunteers, aged between 18 and 50 years of both genders, who have been diagnosed with schizophrenia according to the criteria of the Diagnostic and Statistical Manual of Mental Disorders (DSM-5), through an evaluation conducted via the Structured Clinical Interview for DSM-5 (SCID-5) and who present negative symptoms of moderate intensity (score ≥ 20 on the BNSS). Participants must have stable medication use, with no changes in antipsychotics in the three months prior to inclusion, and must not be using medications that could lower the seizure threshold, except for antipsychotics. Additionally, participants should not have a history of substance dependence or alcohol use in the past four weeks. In the case of smokers, the number of cigarettes and the last use will be documented. Regarding neuromodulation, participants should not have undergone any brain stimulation protocols in the past year prior to study enrollment. All participants must sign the informed consent form to participate in the study.

Participants will be allocated into two groups (intervention and sham), with 30 subjects per group. The statistical power for the primary analysis with this sample size is estimated to be β = 0.82. This calculation assumes (1) the use of mixed linear regression models for hypothesis testing, which will assess the interaction between time (baseline vs. T1) and treatment (intervention vs. sham), (2) a moderate effect size (f = 0.38), and (3) a significance level of 5% (two-tailed).

Randomization will be conducted in blocks of 6 individuals, with permutation in the order and size of the blocks, generated by the website www.randomization.com (accessed on 1 September 2023) and managed by an assistant with no affiliation to the project. Allocation will be carried out using opaque, sealed, standardized envelopes, which will be numbered and assigned to each participant, opened after the individual has been included in the study. The assessing researchers and participants will remain unaware of the type of intervention administered until the study is completed. Protocol implementation will be the responsibility of a researcher who will not collect data and will not be involved in any other aspect of the study, except for protocol application, to ensure blinding.

Exclusion criteria include the following: individuals with comorbid diagnoses of depression or substance use disorder, as defined by the DSM-5, assessed by the SCID; patients who meet DSM-5 diagnostic criteria for organic brain mental disorder or intellectual disability; psychotic episodes or prominent positive symptoms (PANSS positive scale ≥ 20) during the protocol; clinically determined neurological disorders (e.g., epilepsy and a history of seizures or syncope, traumatic brain injury) or severe clinical conditions; the presence of metal implants, such as pacemakers, cochlear implants, implanted electrodes/stimulators, aneurysm clips/coils, stents, or any other criteria common to TMS protocols; any condition that prevents the participant from attending the 5 treatment days.

The study will be conducted at the Integrated Mental Health Care Center Hospital (CAISM) in São Paulo, Brazil.

*Procedures*:

Patients who agree to participate in the study will be included in the sample after signing the Informed Consent Form. The research will be conducted in three phases (Figure 1): Baseline (T0, week 1): During the initial assessment, screening will be performed to verify inclusion and exclusion criteria. Individuals meeting the study criteria will be randomized into the intervention or sham group and will undergo clinical evaluation, heart rate variability (HRV) assessment, and neuropsychological evaluation. Patients must demonstrate clinical stability over the past three months, maintaining a positive psychopathology that is at most moderate (less than or equal to 4 on the items for positive symptoms of the Positive and Negative Syndrome Scale (PANSS) and exhibiting moderate or more severe negative symptoms, defined by scores equal to or greater than 20 on the Brief Negative Symptom Assessment Scale (BNSS). In this phase, baseline HRV will be measured, randomization will be defined, and measurements of the skull cap will be conducted according to the International 10–20 System reference for electroencephalography. Thus, the F3 area (corresponding to the left DLPFC) will be identified using the Beam F3 measurement system [36]. Intensity will be defined by identifying the lowest visible motor response (hotspot), with single pulses on the motor cortex using the lowest power of the equipment, through an automatic measurement system. For this, electromyography, EMG Neuro emg micro-2, (Neurosoft, Ivanovo, Russia)^®^, will be used, with electrodes positioned on the right abductor pollicis brevis muscle. During this phase, the individual will undergo a Safety Inventory for transcranial magnetic stimulation in adults. Intervention and reassessment (T1, week 2): The individual will undergo the protocol, consisting of 20 (twenty) intermittent theta burst stimulation (iTBS) sessions, conducted in 4 daily sessions over 5 consecutive days, i.e., one iTBS session per hour. This protocol corresponds to the total number of sessions conducted by Bation and colleagues; however, it was executed in half the time [16]. The protocol will be conducted with the patient seated in a chair. The coil will be positioned tangentially and perpendicular to the scalp in the area previously marked on a cap. At the end of each treatment day, an Adverse Effects Scale will be used to assess the safety of the technique. Upon completion of the protocol, initial clinical evaluations will be reapplied in the following week, as well as the heart rate variability (HRV) assessment and neuropsychological evaluation. T2 (week 3)—Follow-up 30 days: All assessments will be reapplied 30 days after the intervention.

A pilot study will be conducted at the beginning of the project, in which 10 volunteers will undergo the entire research process in an active protocol, aimed at training the team, adjusting the logistics of the protocol and evaluations, and addressing any physical and psychological aspects related to the volunteers.

### 2.1. iTBS Protocol

The treatment will be conducted using a figure-eight coil positioned over the left DLPFC at a 45-degree angle to the midline, utilizing the Neuro—MS/D device (Neurosoft, Ivanovo, Russia)^®^, controlled by Neuro-MS.NET software (version 3.2.11.0 released 16 July 2023 18:13 x64, Debug), coupled with a rapid recharge module. The intensity will be set at 100% of the observed resting motor threshold, adhering to ethical and safety criteria [37]. The parameters will consist of three pulses at a frequency of 50 Hz, at 5 Hz, using a 2 s train, with an inter-train interval of 8 s, totaling 190 s. Four daily sessions will be conducted hourly, delivering 600 pulses per session. The sham stimulation will replicate the same parameters with coil positioning tangentially to the scalp but will use a sham coil (placebo) to avoid measurable biological effects. The sham coil has an identical appearance to its active version, mimicking its operation; however, it does not produce electromagnetic pulses, thus not promoting a clinical effect. In this way, the subjective somatic sensation on the scalp and the acoustic artifacts during stimulation will be the same for both active and sham volunteers [20,38].

### 2.2. Clinical Assessment

Clinical assessment will be applied three times: at baseline (T0), after the iTBS (T1), and at a 30-day follow-up (T2). The validated Portuguese versions for the Brazilian population of the Brief Negative Symptom Scale (BNSS) [39], and the Positive and Negative Syndrome Scale (PANSS) [40] will be applied to assess negative symptoms and clinical symptomatology. Additionally, the translated and validated versions of the Pittsburgh Sleep Quality Index (PSQI) [41] and the Epworth Sleepiness Scale (ESE) [42] will be used to assess sleep. Generalized Anxiety Disorder-7 (GAD-7) [43] and Clinical Global Impression (CGI) [44], validated instruments for evaluating symptom severity in patients with schizophrenia in Brazil, will also be employed. The Calgary Depression Severity Scale (CDSS) [45] will be applied to check for depressive symptoms. Finally, the Simpson–Angus Scale (SAS) [46] will be administered to identify medication-induced parkinsonian symptoms. All tests will be conducted by trained psychiatrists.

### 2.3. Heart Rate Variability

Data collection of the electrocardiogram (ECG) will be conducted using the Neuro-EMG Micro equipment and PolySpectrum software (Neurosoft, Russia, version 5.3.1.0 released 15 November 2017, with a sampling frequency of 1000 Hz and a 60 Hz notch filter. The patient will remain seated and motionless in a comfortable chair to allow for acclimatization and autonomic stabilization. During this period, the electrodes (RA, LA, LL) will be placed, and signal quality will be continuously monitored. After stabilization, a 5 min baseline will be collected. Subsequently, an additional 7 min of data will be recorded for heart rate variability (HRV) analysis.

The analysis will be performed using Kubios HRV Scientific software (ver. 4.1, Win & Mac, October 2023), calculating measures in the time domain (SDNN, RMSSD, and pNN50), in the frequency domain (VLF, LF, HF, and LF/HF ratio), and sample entropy (SampEn). The factory default settings will be used, including automatic artifact correction at a medium level, automatic beat correction, an interpolation rate of 4 Hz, and a trend removal method. All collections and analyses will adhere to the guidelines set forth by the Task Force of the European Society of Cardiology and the North American Society of Pacing and Electrophysiology (1996).

### 2.4. Neuropsychological Assessment

The Brazilian version of the Measurement and Treatment Research to Improve Cognition in Schizophrenia Consensus Cognitive Battery (MATRICS) is a standardized cognitive assessment for patients with schizophrenia and it will be used to assess changes in cognition. In this study, 9 of 10 subtests from the MATRICS were chosen, given the low-reliability coefficient of MSCEIT in the Brazilian version [39]. We will analyze the following domains: Speed of processing: Trail Making Test: Part A (TMTA); Brief Assessment of Cognition in Schizophrenia (BACS): Symbol Coding and Category Fluency Test: Animal naming (Fluency); Attention: Continuous Performance Test—Identical Pairs (CPT-IP); Working memory: Wechsler Memory Scale—Third Edition (WMS-III): Spatial Span (SS) and Letter-Number Span Test (LNS); Verbal learning: Hopkins Verbal Learning Test—Revised (HVLT-R); Visual learning: Brief Visuospatial Memory Test—Revised (BVMT-R); Reasoning and problems solving: Neuropsychological Assessment Battery (NAB): Mazes. The intelligence coefficient (IQ) will be estimated by the composite score of the subtests of Vocabulary and Matrix Reasoning from the Brazilian Version of the Wechsler Adult Intelligence Scale—3rd edition [47].

### 2.5. Statistical Analysis

The effects of the intervention will be analyzed according to the intention-to-treat (ITT) paradigm, which assesses the effect on all participants, regardless of their level of adherence to the treatment. The primary outcome of the study will be the reduction of negative symptoms, measured by the total score of the Brief Negative Symptom Scale (BNSS) with a score ≥20, while the secondary outcomes will focus on the reduction of negative symptoms as assessed by the negative symptom subscale score of the Positive and Negative Syndrome Scale (PANSS), the sub-domains of BNSS and physiological variables, through HRV assessment, sleep alterations and their association with negative symptoms, assessed by Pittsburgh and Epworth scales, and cognitive assessment by the MATRICS.

Missing data during follow-up will be handled using the restricted maximum likelihood method in mixed-effects linear regression analyses, allowing for the use of all data regardless of whether a participant completes the study, consistent with ITT analysis.

Clinical and demographic differences between groups will be initially evaluated at baseline using the Chi-square test for categorical variables and Student’s *t*-test for independent samples in continuous variables. These same tests will also be employed to identify potential differences between participants who complete the study and those considered to be dropouts. A participant will be considered a dropout if they miss any of the intervention protocol sessions, resulting in a loss of four sessions of intermittent theta burst stimulation (iTBS). A significance level of *p* < 0.05 will be adopted in all statistical analyses.

Differences between the baseline, after intervention, and follow-up (T0, T1, and T2) for each measure will be tested using mixed-effects linear models. The treatment effect will be estimated through the interaction between the intervention group (intervention or sham) and the assessment time (T0, T1, and T2). Effect estimates will be presented as post-intervention mean differences, along with 95% confidence intervals, *p*-values, and Cohen’s d effect sizes (where 0.2 represents a small effect, 0.5 represents a moderate effect, and 0.8 represents a large effect). Random effects from repeated measures within each subject will be modeled according to the covariance structure that shows the lowest index based on the Akaike information criterion (AIC). The following covariates will be included in the models: the Calgary Depression Severity Scale score, as depressive symptoms may confound the assessment of negative symptoms and individuals with severe depression will be excluded from the sample; the total score of the Clinical Global Impression-Severity scale (CGI-S), since the severity of illness may influence treatment response; and the Generalized Anxiety Disorder 7-item scale (GAD-7), due to anxiety symptoms. For the various exploratory analyses conducted with the previously detailed secondary outcomes, the significance value will be corrected for multiple tests using Bonferroni criterion, with an alpha of 0.05 divided by the number of tests.

## 3. Discussion

This study aims to evaluate the effects of an accelerated iTBS protocol on negative symptoms of schizophrenia, aiming to establish an effective and feasible protocol for clinical application. To achieve this, clinical assessments will be utilized, along with their association with cognitive aspects, sleep, and heart rate variability (HRV), which will be fundamental for understanding the interaction between the clinical and biological aspects involved.

Recent studies have shown the therapeutic potential of TMS for negative symptoms in schizophrenia. A meta-analysis study that included a total of 2633 participants indicates the efficacy of this therapeutic modality, especially when the target of intervention is the left dorsolateral prefrontal cortex (DLPFC). However, there is a high risk of bias due to substantial heterogeneity among the included studies [35]. These results were corroborated by a comparative review study of non-invasive neuromodulation techniques. One justification for this could be the underlying mechanism of action of the left DLPFC on negative symptoms, as well as its relationship with other structures implicated in these symptoms, such as the midline of the cerebellum [48]. This reinforces the premise of the efficacy of repetitive TMS across distinct symptom domains rather than focusing on specific diagnoses [49]. On the other hand, a randomized, double-blind, placebo-controlled, multicenter clinical trial involving 175 patients with schizophrenia and predominantly negative symptoms did not observe the efficacy of active treatment with 15 sessions of high-frequency rTMS on the left DLPFC. One possible reason for the non-significant result was the relatively small number of sessions [50].

In this context, accelerated protocols (two or more TMS sessions in a single day) emerge as a safe and effective alternative, capable of substantially reducing the total treatment time. Theta burst stimulation (TBS), which delivers TMS pulses in bursts at gamma frequency (e.g., 50 Hz) repeated at theta frequency intervals [51], has been utilized in this type of protocol due to its short session duration [52]. This technique has increasingly been used in the treatment of psychiatric disorders [53], since its inception as a powerful means of inducing neuroplasticity [9,54]. This is evidenced by an open study by Cole et al., 2020 [13], in which patients with treatment-resistant depression achieved a remission rate of approximately 90% in just 5 days of treatment. This type of protocol may influence the adjustment of factors that directly affect treatment response, such as the number of sessions per day, total session duration, the inter-session interval, and the total number of sessions in TMS treatment [12]. The efficacy of treatment with iTBS appears to be dose-dependent, as previously observed, with a more favorable intervention effect seen in patients who received stimulation with ≥1800 pulses per day over a total of ≥20 sessions [55].

Considering studies that have utilized iTBS for negative symptoms of schizophrenia, there is still considerable uncertainty in treatment, particularly regarding variations in the type of protocol and the number of sessions, as well as the brain region to be stimulated. When comparing the efficacy of different high-frequency TMS protocols (10 Hz and 20 Hz) with iTBS in treating prominent negative symptoms, the TBS group showed significantly greater reductions in these scores [56]. A recent meta-analysis that included 12 studies with iTBS intervention in participants with prominent negative symptoms revealed a larger effect size favoring iTBS, especially in cases where the targets were aimed at the left DLPFC (compared to cerebellar targets), as well as greater effects in symptom relief for protocols with a higher total number of iTBS pulses [11]. Although a single iTBS has shown promise, accelerated protocols with longer intervals (60 min) between sessions using this technique appear to improve outcomes through additive metaplasticity, preventing homeostatic metaplasticity from stabilizing the effects of stimulation [52].

Other parameters, such as cognition, sleep, and HRV assessment, may broaden the findings of the study, contributing to an understanding of the mechanisms of improvement in the treatment and its potential relations to the nervous system. A meta-analysis of the effects of iTBS in healthy subjects suggests that a single session improved cognition with a small effect size [57]. In schizophrenia, neuromodulation on the left DLPFC by iTBS was associated with improved visuospatial working memory after 14 sessions (using a 3-back task) [58] and enhanced social cognition after 20 sessions [18]. Considering these initial findings, our hypothesis is that an accelerated protocol will impact working memory functions, specifically visuospatial working memory, measured by the Matrics span task.

Regarding sleep, the activity of the prefrontal cortex—a crucial brain area for cognition, emotion, and behavior, including sleep regulation—could be enhanced by iTBS, potentially increasing cortical activity and improving functional connectivity, which is a measure of communication between different brain areas. For instance, an improvement in sleep quality might be associated with a reduction in negative symptoms. Moreover, sleep assessment may help identify potential treatment responders, serving as a predictor of therapeutic response [59].

Furthermore, exploring the impact of neuromodulation on HRV will contribute to filling a gap in the literature regarding the link between DLPFC functioning and autonomic nervous system regulation in schizophrenia. Resting vagal activity in people with schizophrenia, measured through HF and RMSSD variables, is lower compared to healthy individuals [25] and unaffected first-degree relatives [60], as well as HRV complexity (Poincaré measures), indicating impaired physiological adaptation in recovery after stress [61].

Ma et al. observed that patients with greater insight impairment (as measured by the G12 PANSS variable) present lower HF values, poorer medication adherence, and greater working memory deficits. However, the relationship between autonomic impairments, cognitive functioning, and negative symptoms in individuals with schizophrenia remains underexplored [62]. Although research on this topic is still limited, DLPFC stimulation via rTMS has resulted in increased HRV, enhanced cardiac vagal control, and reduced arousal in autism [63] and in the modulation of the fronto-vagal network in depression [64]. In schizophrenia, no study has explored HRV modulation after iTBS, nor its association with negative symptoms and cognition and the association between HRV, negative symptoms in schizophrenia and sleep quality. HRV emerges as a measurable biomarker that, in addition to investigating the relationship between the autonomic nervous system and the clinical profiles of patients, particularly those with predominant negative symptoms, facilitates the exploration of response indicators and potential predictors of the effects of neuromodulation.

In summary, the effects of the accelerated iTBS protocol on negative symptoms have been investigated with promising perspectives; however, there is a need to establish a protocol that is both effective and feasible, particularly regarding high dosage. Although some protocols of this nature demonstrate efficacy, logistical barriers often compromise clinical application, complicating patient adherence. In this accelerated protocol, the patient will spend a short period daily (just over 3 h), which may indeed enhance feasibility. Furthermore, this project provides a platform to deepen the understanding of the effects of neuromodulation on physiology, contributing to the comprehension of the underlying mechanisms involved.

## 4. Limitations

We will not utilize functional neuronavigation as a method for targeting stimulation, which is considered the most precise technique for this type of intervention, due to practical and logistical limitations. Instead, we will employ the Beam F3 technique based on the international 10–20 system used in electroencephalography. This technique is validated for such interventions and is widely used. Regarding the blinding of the study, the applicator will not be blinded to the intervention, as the equipment requires changing the coils depending on the type of intervention (active or sham). This issue will be minimized by ensuring that the applicator does not have contact with the evaluations and assessors involved in the study.

## 5. Conclusions

This study is a randomized, double-blind, sham-controlled clinical trial comparing groups of subjects with negative symptoms of schizophrenia. It will investigate the effects of an accelerated iTBS protocol on the left DLPFC through clinical assessments, a neuropsychological battery, and heart rate variability measurements. It is expected that the results of this study will contribute to the optimization of therapeutic protocols for this disorder, particularly concerning clinical applicability.

## Data Availability

Not applicable.

## Abbreviations

The following abbreviations are used in this manuscript.

iTBS	Intermittent theta burst stimulation
NS	negative symptoms
BNSS	Negative Symptom Scale
NIBS	Non-invasive brain stimulation
TMS	Transcranial Magnetic Stimulation
DLPFC	Dorsolateral prefrontal cortex
HRV	Heart rate variability
PANSS	Positive and Negative Syndrome Scale
PSQI	Pittsburgh Sleep Quality Index
ESE	Epworth Sleepiness Scale
GAD-7	Generalized Anxiety Disorder-7
MCCB	MATRICS Consensus Cognitive Battery
CDSS	Calgary Depression Severity Scale
SAS	Simpson–Angus Scale
DSM-5	Diagnostic and Statistical Manual of Mental Disorders
SCID-5	Structured Clinical Interview for DSM-5
ECG	Electrocardiogram
AIC	Akaike information criterion
CGI-S	Clinical Global Impression-Severity scale
LF	Low frequency
HF	High frequency
RMSSD	Root mean square of consecutive differences
EMG	Electromyography
